# Improving Public Health Agency and System Performance: Fortification for Promoting Population Health and Wellness

**DOI:** 10.5888/pcd10.130202

**Published:** 2013-07-18

**Authors:** Judith A. Monroe, Craig Thomas

**Affiliations:** Author Affiliation: Judith A. Monroe, Office for State, Tribal, Local, and Territorial Support, Centers for Disease Control and Prevention, Atlanta, Georgia.

America faces a new frontier in preventing chronic disease. Nearly 80% of the 10,000 people who turn 65 each day have at least 1 chronic health condition, and most have multiple chronic conditions (1). The costs of braving this new world are staggering, especially given the nation’s strained economy and budget cuts that have forced health departments to reduce their workforce and impose furloughs and reduce or eliminate chronic disease programs. Despite these daunting challenges, government public health agencies have opportunities to be the driving force behind improving the nation’s health.

The Office for State, Tribal, Local, and Territorial Support (OSTLTS) at the Centers for Disease Control and Prevention (CDC) was established in 2010 to better position CDC to support health departments. The mission of OSTLTS is to advance US public health agency and system performance, capacity, agility, and resilience (2). OSTLTS recognizes health departments as the front line of prevention and promotes a systems approach that works across sectors, partners, and programs to address the complex community and economic challenges that affect the public’s health.

An important and recent advancement in the practice of public health is the establishment of a national public health accreditation program and the Public Health Accreditation Board (PHAB). Funded by OSTLTS and the Robert Wood Johnson Foundation, PHAB seeks to advance the quality and performance of public health departments through national standards, which more than 3,000 health agencies can use to continuously improve performance. Based on the 10 Essential Public Health Services (3), PHAB accreditation offers a means to ensure comprehensive and quality programs for a range of public health areas, including chronic disease prevention. In March 2013, the first 11 public health departments were accredited by PHAB, and many more applications are in process (4).

To support accreditation readiness, OSTLTS launched the National Public Health Improvement Initiative, which currently provides 73 state, local, territorial, and tribal health agencies with funding and assistance to prepare for accreditation and to strengthen agencies’ performance management and quality improvement capacity (5). Each grantee is required to hire or identify a performance improvement manager, thus creating a national network of performance improvement managers. The National Public Health Improvement Initiative is the first noncategorical cooperative agreement at CDC that supports assessment against the PHAB standards to address critical gaps in service delivery and the implementation of quality improvement methods and practices for greater program efficiency and effectiveness.

National Public Health Improvement Initiative funding has focused on several chronic disease program areas, including tobacco-use prevention; healthy living (obesity, physical activity, and nutrition); healthy aging (heart disease, stroke prevention, and hypertension); diabetes; cancer prevention; and asthma. For example, the Philadelphia Public Health Department created the first citywide estimate of hypertension based on local health care data rather than sample survey data; New York City added more than 500 clinical practices to its information system, increasing data and information reporting on diseases and conditions such as diabetes, hypertension, obesity, and cancer. Montana is developing a colorectal cancer screening toolbox, and it has improved coordination between medical care and home and school environments through its asthma home visiting program (5).

To understand and address public health’s most pressing challenges, OSTLTS works closely with a state, tribal, local, and territorial workgroup that makes recommendations to the Advisory Committee to the Director (6). The workgroup has recommended ways for CDC to better support health departments in the future, and OSTLTS, along with the offices of the Associate Director for Program and the Associate Director of Policy, oversees the implementation and adoption of the advisory committee recommendations. One of the first workgroup recommendations was to improve the technical assistance and services delivered to state, local, tribal, and territorial grantees by CDC project officers. In response, OSTLTS initiated the Technical Assistance and Services Improvement Initiative, which provides agency-wide training, information, and support to project officers. OSTLTS also administered to field staff the first-ever survey, which focused on improving grants management, project officer competencies, and types of assistance needed to enhance program operations and improve health outcomes. Other system-wide improvements include the development of a standard template for all new funding opportunity announcements to reduce inconsistency in the development of new announcements and strengthen program accountability to funders and the field.

Other recommendations touch on the future of public health with a focus on promoting the intersection of clinical medicine and public health. In early 2012, OSTLTS launched the Primary Care and Public Health Initiative. This initiative is designed to make it easier for residency program faculty members to incorporate population health into the curriculum. It also provides up-to-date public health data for evidence-based decision making.

OSTLTS recognizes that the success of public health programs and chronic disease programs is grounded in a well-trained and skilled workforce. To enhance the workforce, OSTLTS was charged with the growth and development of the Public Health Associate Program, which trains college graduates to be the public health advisors of the future. The curriculum and competencies for this program have been carefully crafted according to the needs of the future workforce; the program will continue to be flexible and dynamic. Health departments can apply to be a host site for an associate’s 2-year training. Many of the associates in this program have been assigned to chronic disease as their area of focus. As an example, a 2011 associate working in New Orleans searched for grant opportunities to sustain the Fit NOLA Partnership, which aims to make New Orleans one of the United States’ 10 fittest cities by 2018 (7). The City of New Orleans Health Department received grants from Blue Cross and Blue Shield of Louisiana, the Partnership for a Healthier America, and the Robert Wood Johnson Foundation as a result of the associate’s work.

Finally, OSTLTS sees leadership development as crucial to responding effectively to the challenges inherent in a changing public health landscape. OSTLTS designed an applied multisector leadership development model for the National Leadership Academy for the Public’s Health, which has been implemented by the Public Health Institute through a competitive cooperative agreement with CDC (8). Focused on community-level projects, the academy will train several Community Transformation Grant leadership teams, with a focus on addressing problems in preventing chronic diseases at the local level.

Change is difficult, requiring strong leadership, perseverance, and openness to new ideas. As health reform evolves, systems thinking and multisector engagement will continue to be priorities for OSTLTS and will serve as critical components of our efforts to improve the health and wellness of Americans.
